# Prevalence and Risk Factors for Portal Cavernoma in Adult Patients with Portal Vein Thrombosis

**DOI:** 10.3390/diagnostics14131445

**Published:** 2024-07-06

**Authors:** Sergiu Marian Cazacu, Dragoș Ovidiu Alexandru, Daniela Dumitrescu, Alexandru Marian Vieru, Marinela Cristiana Urhuț, Larisa Daniela Săndulescu

**Affiliations:** 1 Research Center of Gastroenterology and Hepatology, Gastroenterology Department, University of Medicine and Pharmacy of Craiova, Petru Rares Street no. 2-4, 200349, Craiova, Romania; cazacu2sergiu@yahoo.com (S.M.C.); larisasandulescu@yahoo.com (L.D.S.); 2 Biostatistics Department, University of Medicine and Pharmacy of Craiova, Petru Rares Street no. 2-4, 200349 Craiova, Romania; dragosado@yahoo.com; 3 Imaging Department, University of Medicine and Pharmacy of Craiova, Petru Rares Street no. 2-4, 200349 Craiova, Romania; daniela.dumitrescu@gmail.com; 4 Doctoral School, University of Medicine and Pharmacy of Craiova, Petru Rares Street no. 2-4, 200349 Craiova, Romania; alexandruvieru1993@gmail.com

**Keywords:** portal vein thrombosis, portal cavernoma, hepatocellular carcinoma, contrast-enhanced ultrasound, thrombophilia

## Abstract

Portal vein thrombosis (PVT) represents a restriction or occlusion of the portal vein by a blood clot, which can appear in liver cirrhosis, inherited or acquired thrombophilia, malignancies, abdominal infection, abdominal inflammation, and injury to the portal vein; it can evolve to local venous extension, recanalization, or portal cavernoma (PC). This research represents an observational study of patients admitted with a diagnosis of PVT between January 2018 and December 2022. We assessed the rate of and risk factors for PC. In total, 189 patients with PVT were included; the rate of PC was 14.8%. In univariate and multivariate analysis, the main risk factors for the presence of PC were etiology (thrombophilia, myeloproliferative disorders, local inflammatory diseases, and idiopathic causes), prior PVT, and complete versus incomplete or single-branch portal obstruction. In patients with superior mesenteric vein (SMV) thrombosis, distal obstruction was more prone to PC than proximal obstruction. The main predictive factors were etiology, prior PVT, complete PVT obstruction, and no prior non-selective beta-blocker (NSBB) use; in patients with SMV thrombosis, the distal extension was more significantly associated with the risk of PC. We propose a composite score for the prediction of PC which includes etiology, prior diagnosis of PVT, prior NSBB use, complete versus incomplete PVT, and distal versus proximal SMV thrombosis, with good accuracy (AUC 0.822) and an estimated sensitivity of 76.92% and specificity of 82.39% at a cut-off value of 4.

## 1. Introduction

Portal vein thrombosis (PVT) represents a restriction or occlusion of the portal vein by a blood clot [1,2,3,4,5], which can appear in liver cirrhosis, inherited or acquired thrombophilia, malignancies (solid tumors and myeloproliferative syndrome, especially in policitemia vera and essential thrombocytopenia), abdominal infection, abdominal inflammation, and injury to the portal vein (Table 1). A classification of cirrhotic, malignant, and non-cirrhotic non-malignant PVT is frequently used [4]. The disease is rarely encountered in post-mortem studies (0.05 to 1%) [1,3] but is estimated at 5–26% of liver cirrhosis patients awaiting liver transplantation, in up to 40% of cases of hepatocellular carcinoma (HCC), and a significant proportion of cases (especially in cirrhotic patients) are asymptomatic [1,6,7,8]. In patients with liver cirrhosis, PVT is estimated at 3.6% in primary sclerosing cholangitis, 8% in primary biliary cirrhosis, and 16% in alcoholic and hepatitis B virus-related cirrhosis [9].

Portal stasis, together with local and systemic hypercoagulability factors and abdominal trauma (including surgery) represent the main factors for PVT [1,2,4]. PVT may appear in the presence of cirrhosis and/or HCC or in patients without liver disease. In patients with cirrhosis, portal stasis represents the main mechanism [2,3,6,7,8], because of increased intrahepatic vascular resistance and decreased velocity of the portal flow, although hypercoagulability status is also often encountered (increased factor VIII levels, decreased protein C level, factor V Leyden G1691A, methyl-tetrahydrofolate reductase C677T and prothrombin G20210A mutations, and antiphospholipid antibodies) [1,3,6,7,8]. In stable cirrhosis with PVT, thrombophilic factors may be incriminated in PVT appearance [1], while in advanced cirrhosis endotoxinemia is frequently encountered and may activate local clotting mechanisms with potential increased risk for PVT, and endothelial damage following sclerotherapy was also incriminated in some studies [6]. In up to 70% of patients with cirrhosis and PVT, a concomitant systemic risk factor for thrombosis may be detected [2].

PVT may appear in the context of local or distant malignancy and also in myeloproliferative disorders and other hematological malignant diseases [1,2,4]. Direct compression (bland thrombosis) or invasion (tumor thrombosis) represent the main mechanisms for PVT in local malignancies, although the hypercoagulable state may be an additional factor [2,3]. Imaging procedures, such as contrast-enhanced ultrasound (CEUS), MRI, CT scan, or 18F-FDG PET, can help to differentiate between bland and tumor thrombosis, and a score (A-VENA) which incorporates alpha-fetoprotein level and imaging characteristics of the tumor (venous expansion; thrombus enhancement; neovascularity; and adjacent position to HCC) was also proposed [10].

The clinical presentation depends on the etiology, thrombus location, the degree of portal occlusion, and the extension to the superior mesenteric vein or splenic vein [1]; the acute form is usually symptomatic, while the chronic form (especially in cirrhosis) can be asymptomatic or symptomatic [1,2,6,8]. Acute PVT is defined as symptoms onset <60 days before the diagnosis with no portal cavernoma (PC) or portal hypertension signs [11]. PVT can evolve to local venous extension, total or partial recanalization, or can be followed by the appearance of portal cavernoma, which is a vascular neoformation or interconnection of collateral vessels proximal and distal to the thrombus [1,12,13,14], as early as 6 days [11,15,16] but usually 3 to 5 weeks after acute onset [1,3,14]. Transient PVT in cirrhosis with spontaneous recanalization was also described [17]. In children, PC may be congenital or may appear as a complication of PVT [18]. A significant proportion of PVT and PC has no identified cause; many “idiopathic” cases may be explained by uninvestigated prothrombotic factors [19]. In some studies, 30 to 50% of PC cases were idiopathic [16,20,21,22]. In some cases, PC may appear at the onset without prior PVT and, rarely, as an onset of myeloproliferative disease [5,23,24]. The development of PVT is not associated with unfavorable prognosis in liver cirrhosis [25]. In contrast, in cases associated with malignant diseases, the prognosis is related to primary malignancy [4], and in HCC the appearance of PVT may significantly alter the prognosis [3].

The development of PC is frequently insufficient for the normalization of hepato-petal blood flow and complications related to portal hypertension can occur. Acute variceal bleeding is more frequent in children than in adults, and is associated with a lower mortality rate because liver function is conserved; ectopic varices and collateral circulation development with subclinical encephalopathy were also noted [3,4]. Transient ascites and splenomegaly with hypersplenism were present. Intestinal congestion or ischemia can appear if the mesenteric vein is involved [1,3,4]. Portal biliopathy developed in up to 80–100% of chronic PVT [1,2,3,4], most cases being asymptomatic [1,3,4].

**Table 1:** Localized and systemic causes of PVT [1,2,3,6,16,21,26,27,28].

	Category	Diseases
Localized	Liver cirrhosis (28%)	
	Malignancy (27–44%)	Hepatocellular carcinoma Gastric carcinoma Pancreatic carcinoma Cholangiocarcinoma Lymphoma
	Local inflammation (10%)	Pancreatitis Cholecystitis Appendicitis Inflammatory bowel disease Diverticulitis Acute hepatitis (including cytomegalovirus) Perforation
	Portal vein injury	Abdominal surgery Local trauma Shunts Liver transplantation
Systemic	Inherited thrombophilia	Factor V Leyden mutation Prothrombin gene mutation Protein C, S, and antithrombin III deficiency Methylenetetrahydrofolate reductase (MTHFR) mutation Hyperhomocysteinemia
	Acquired thrombophilia	Myeloproliferative disorders Antiphospholipid syndrome Paroxysmal nocturnal hemoglobinuria
	Other	Pregnancy, oral contraceptives
Idiopathic		

The PVT classification is made using three criteria (degree, extension, and acute or chronic onset) [1,2,3,4]. By the degree of the obstruction, PVT can be classified as incomplete (partial obstruction, with a residual lumen with blood flow present) or complete [3,9]. Using the extension criteria, PVT can be classified (Yerdel classification) as grade I, with thrombus less than 50% of the lumen with no or minimal extension to the superior mesenteric vein (SMV); grade II, with a thrombus in more than 50% of the lumen with no or minimal extension to the SMV; grade III, with complete PVT extending to the proximal but no distal SMV; and grade IV, with complete PVT and complete thrombosis of the SMV [1,6,22]. Another study has proposed a classification based on the degree of the obstruction and the presence of the cavernoma: type I, with incomplete obstruction and no PC; type II, with incomplete obstruction with PC; type III, with complete obstruction and no PC; and type IV, complete obstruction with PC [11].

**Figure 1:**
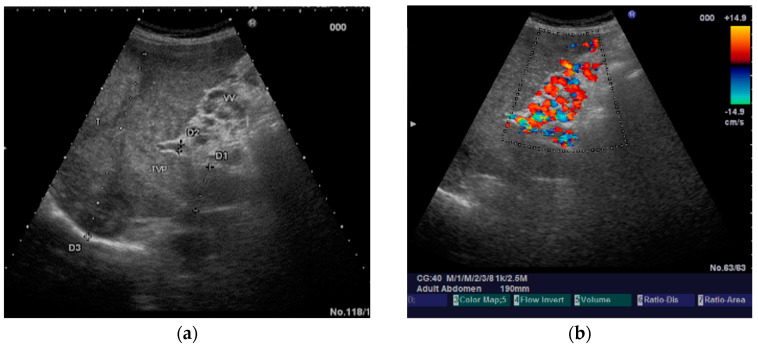
Portal cavernomatous transformation. (**a**) Multiple serpiginous echo-free structures (vessels) replacing the normal portal vein are seen in B-mode at the hepatic hilum; (**b**) Color Doppler examination confirms the presence of venous flow within the vessels of the cavernoma.

**Figure 2:**
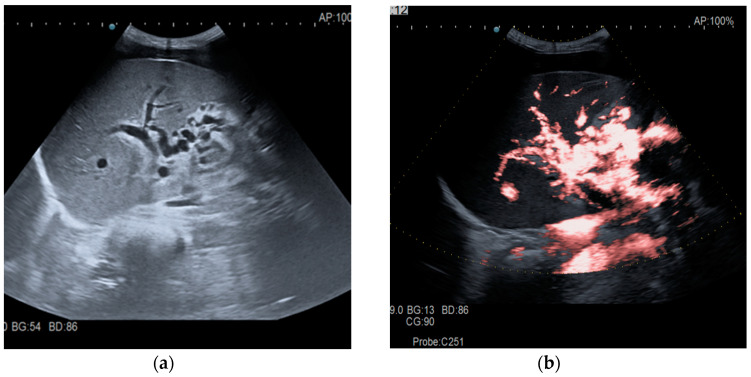
Portal cavernomatous transformation. (**a**) Portal vein cannot be identified; instead, meandering venous branches are visible in the porta hepatis, indicating portal vein thrombosis and consequent cavernous transformation. (**b**) Power Doppler ultrasonography identifies flow within the vessels.

**Figure 3:**
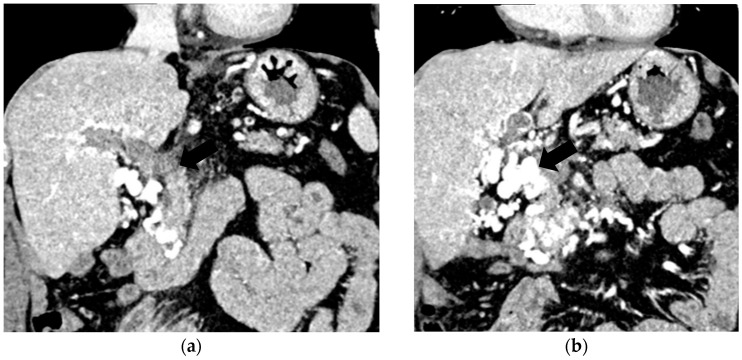
CT scan in a patient with recent surgery for transverse colon adenocarcinoma. (**a**) The enlarged portal vein, with intraluminal thrombus and peri-gastric collateral circulation, indicated by black arrow. (**b**) A 59/34 mm portal cavernoma, indicated by black arrow.

**Figure 4:**
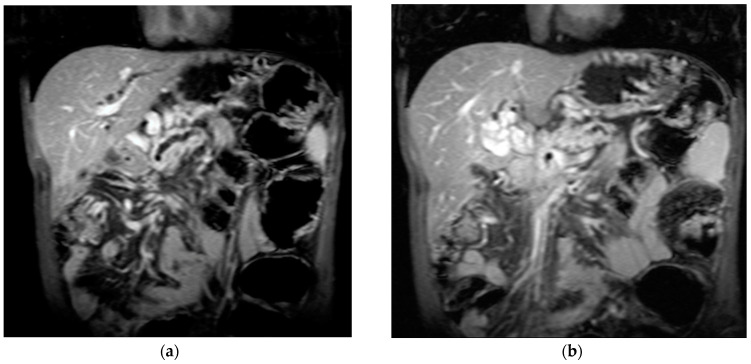
MRI scan in a patient with pancreatic carcinoma. (**a**) Enlarged portal vein, with intraluminal thrombus. (**b**) A hilar 5.1/3.3 cm portal cavernoma.

**Figure 5:**
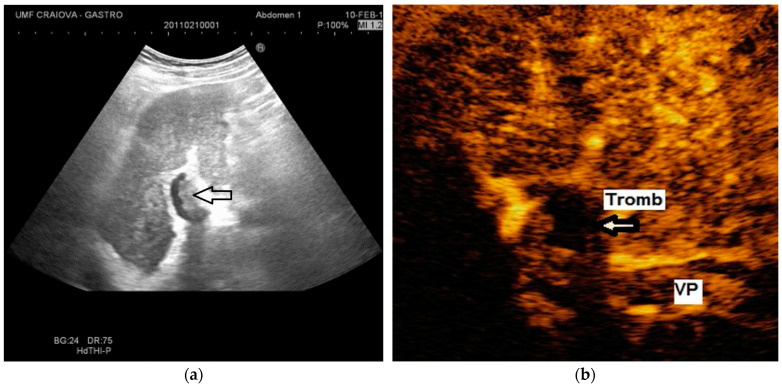
Benign portal vein thrombosis. (**a**) B-mode image demonstrating incomplete echogenic material within the lumen (arrowhead). The thrombus at the main portal vein affects less than 50% of the lumen. (**b**) No contrast uptake is observed in CEUS (arrowhead).

**Figure 6:**
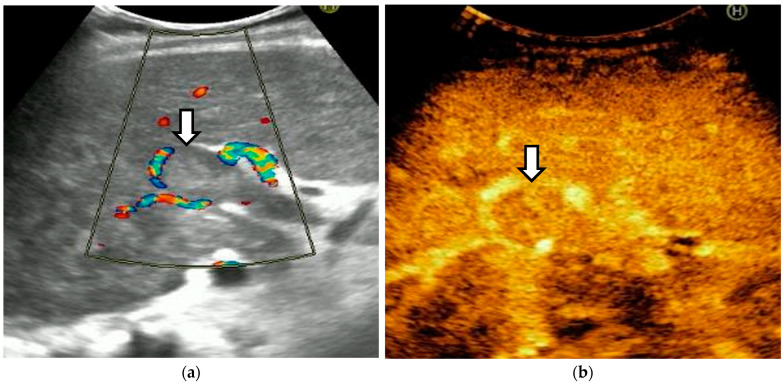
Malignant portal vein thrombosis. (**a**) In standard ultrasound, the portal vein is large with an occlusive thrombus (arrowhead). (**b**) CEUS reveals enhancing tissue within the vessel lumen (arrowhead) in the arterial phase.

The diagnosis of PVT and PC is made by imaging procedures (transabdominal ultrasound with a Doppler or contrast-enhancing computed tomography scan, magnetic resonance imaging, or angiography in the case of therapeutic intended procedures) [1,3,12,13,14,21,29]. Transabdominal ultrasound is a reliable procedure in 60–100% of cases, with an anechoic aspect in recent cases (requiring Doppler examination or CEUS) and a hypoechoic or hyperechoic aspect in chronic cases [22,29]. Doppler examination may show an anechoic or hypoechoic thrombus and a slower (<15 cm/sec) or absent portal blood flow, and is considered the gold standard (color Doppler) for the diagnosis of PC [1], with a sensitivity and specificity of 95% (Figure 1 and Figure 2) [22]. CEUS can help the diagnosis of benign or malignant PVT, can characterize associated focal liver lesions [30], allows a better characterization of PVT [31], and also permits better detection of PC—Figure 3 and Figure 4 [32]. CT scan and MRI (Figure 5 and Figure 6) are more accurate for the evaluation of liver causes (HCC, abscesses, and other tumors) or other local causes (pancreatitis, diverticulitis, and appendicitis), and for complications (bowel infarction or perforation), and may show permeability of the portal venous system and the flow direction [1,22]. Malignant PVT has intra-thrombus arterial signals on CT, MRI, and/or CEUS, with portal vein diameter frequently 23 mm or above, and with the presence of the tumor at imaging examination (Figure 4 and Figure 6) [21].

The purpose of this study was to assess the prevalence of and predictive factors for the development of PC in patients with PVT. Although a significant proportion of PVT patients develop PC, the rate of PC detection in PVT patients is poorly evaluated in the literature; most studies consider only the degree of obstruction and acute or chronic form as risk factors [9,14]. The etiology of PVT may also be important because the initial portal pressure is higher in liver cirrhosis and also in HCC associated with liver cirrhosis; also, the extrahepatic and intrahepatic collateral circulation is already present in cirrhotic patients. The influence of NSBBs can also be an important factor [6,7,8].

## 2. Materials and Methods

We performed an observational study to analyze the prevalence and risk factors for PC in patients with PVT admitted to the Emergency County Hospital Craiova (a tertiary academic hospital) for 4 years (January 2018–December 2021). Data from patients were obtained from the Hippocrate computerized system of the hospital and by analyzing the medical records of the patients; we selected patients aged 18 years and older admitted for acute or chronic PVT coded by ICD-10-AM as portal vein thrombosis (I81), portal vein phlebitis (K75.1), and aberrant portal venous connection (Q26.1). Informed consent was obtained from all patients admitted and approval of the Local Ethical Committee of the Emergency County Clinical Hospital Craiova was also obtained (43331/05.09.2023).

The diagnosis of PVT and PC was made by either CT scan, MRI, or transabdominal ultrasonography with Doppler examination or CEUS. PVT was assessed as acute (symptoms onset within less than 60 days) or chronic and complete or incomplete. SMV and splenic vein thrombotic extension was assessed by CT scan or MRI. Patients with PVT were divided by etiology into four categories: cirrhotic PVT, HCC-PVT, other malignant PVT (excluding HCC), and other PVT. PC was diagnosed by CT scan, MRI, or Doppler US/CEUS and was assessed as absent or present. The etiology of cirrhosis was also noted.

Two CT scan equipments were used: CT Siemens Somatom go TOP 128 slices reconstructed (Siemens Healthineers, Erlangen, Germany) and CT Scan GE Revolution 128 slices reconstructed (GE HealthCare Technologies, Inc, Chicago, Illinois, USA). For IRM two devices were also used: RM Siemens—Magnetom Symphony 1,5T ((Siemens Healthineers, Erlangen, Germany), and RM GE Signal Explorer 1,5T (GE HealthCare Technologies, Inc., Chicago, Illinois, USA).

CEUS was performed by using a Hitachi-Aloka Preirus Arietta 70 (Tokyo, Japan). Contrast-enhanced imaging bearing low-mechanical-index cadence contrast pulse sequencing technology using an ultrasound contrast agent (UCA) SonoVue (Bracco, Geneva, Switzerland) was employed.

The phases of contrast enhancement were considered as follows: arterial phase acquired between 10 and 30 s after contrast injection, portal venous phase between 31 and 120 s, and late phase if it was detected after 120 s. All video clips were stored for later review.

The assessment of the arrival time of the UCA to the portal vein was carefully evaluated. If the portal vein thrombus was “avascular”, then it was characterized as an acute bland thrombus (benign thrombus). Any thrombus that showed the same enhancement characteristics as the tumor from which it originated, including rapid APHE (Arterial Phase Hyperenhancement) and washout, was defined as “tumor-in-vein” (malignant thrombus).

All CEUS exams were acquired by the same gastroenterologist, who was experienced in CEUS (EFSUMB level 3 sonographer, with more than 10 years of experience in CEUS (>6000–10,000 ultrasound examinations)).

Statistical data were analyzed and provided using MedCalc version 22.009 (MedCalc Software Ltd., Ostend, Belgium) and XLSTAT 2020.3.1 (Addinsoft. SARL, Paris, France). Numerical variables were compared using Student’s *t*-test, for two groups, or ANOVA, for three or more, because they were found to have a normal (Gaussian) distribution within each group, using the Shapiro–Wilk normality test, while, for categorical variables, the Chi-square test was used. To assess the risk of PC, coded as a binary variable, we used the variables with a significant impact detected for the univariate analysis in a multivariate logistic regression model. Based on the OR (Odds Ratio) obtained in this model, we tried to develop a scoring system, as it would be easier to use in practice.

## 3. Results

### 3.1. General Characteristics of Patients with Portal Vein Thrombosis

The mean age was 63.2 ± 11.3 years (most patients between 40–79 years). Of the patients, 72% were males. The etiology of PVT was dominated by tumors (54.5%) and liver cirrhosis (29.1%). The presence of cavernoma was recorded in 14.8%; twenty-four cases appeared at the moment of the diagnosis and four cases during a median follow-up of 22 months. Acute onset (with abdominal pain) was noted in 19% of patients; onset with UGIB was recorded in 17.4%. The extension of the thrombus in SMV was recorded in 25%, and in SV in 35.1% (Table 2).

Transabdominal ultrasound with Doppler protocol (Figure 1a,b and Figure 2a,b), CT scan (Figure 3a,b), and MRI (Figure 4a,b) were used for the diagnosis in 88.9, 75.1, and 15% of cases, respectively. CEUS was used for the diagnosis in 49 cases (25.9%) but in only 29 cases was it used for the evaluation of the thrombus, and in two cases for cavernoma diagnosis (Figure 5a,b and Figure 6a,b).

CEUS detected contrast uptake in none of 10 benign thrombi (100%) and 17 of 19 (89.47%) malignant thrombi. The two unenhanced malignant thrombi were small and incomplete. All the malignant thrombi exhibited early blood flow (arterial phase, up to 30 s after contrast injection). Malignant thrombi had the same enhancement pattern as the tumor from which they originated, including rapid arterial phase hyperenhancement and slow or weak portal venous washout. In 20 cases, we visualized small hyperechoic portal vein lumen, which reflects an old thrombosis with or without PC.

Endoscopy was performed in 108 cases and showed the presence of esophageal varices in over 80% of cases, mostly related to cirrhosis presence.

### 3.2. Portal Cavernoma Patients and Patients with PVT but No Portal Cavernoma

Next, we divided patients with PVT into two groups: those with PC and those with no PC; their characteristics are presented in Table 3.

There was a significant statistical difference regarding age between patients with PC and those with no cavernoma (mean difference 6.85, 95%CI 2.3799 to 11.3157, *p* = 0.003). Gender prevalence showed a predominantly male gender in both groups (66.7% in the PC group and 72.8% in the non-PC group, *p* = 0.330). In the PC group, we noted fewer patients with HCC and cirrhosis and more patients with other etiology such as inflammatory diseases, thrombophilia, and pancreatitis (*p* < 0.001). Prior PVT was noted in 17.9% of patients with PC and 5% of patients without cavernoma (OR 4.15, 95%CI 1.25 to 13.80, *p* = 0.02); rates of prior UGIB were similar (7.1 and 3.7%, OR = 0.95, 95%CI 0.11 to 8.26, *p* = 0.952). Prior use of NSBBs was noted predominantly in the non-PC group (61.5 versus 35.7%, OR = 2.87, 95%CI 1.24 to 6.62, *p* = 0.013); the proportion of prior anticoagulant use was similar (14.3 and 9.9%, respectively, *p* = 0.493). The rate of acute onset with abdominal pain was similar in both groups (14.3 and 19.9%, respectively, *p* = 0.489); onset with acute UGIB was also similar (14.3 and 17.4%, *p* = 0.952).

In the PC group, more patients had complete PV obstruction than incomplete or one-branch obstruction (*p* = 0.0005), and more patients had distal than proximal SMV thrombosis; the presence and the type of SV obstruction were similar.

Next, we performed a multivariate analysis to assess which parameters were independently associated with the risk of PC presence in our group of patients (Table 4). To properly assess the independent parameters, all factors with a statistical *p*-value of less than 0.25 regarding the difference were introduced into the initial model.

Because we found a significant relationship between age and etiology, and also a highly significant relationship between etiology and the use of beta-blockers (*p* Chi-square = 0.00000017 < 0.001) we decided to create a model using only four variables (Table 5), which increases accuracy from 88.69% to 89.29%.

Because multivariate logistic regression models are hard to use in everyday practice, based on data obtained by the univariate and multivariate analysis, we proposed a score composed of the statistically significant risk factors previously identified, trying to assign different values to the categories of the variables employed based on the OR values obtained in the multivariate logistic regression models.

We decided on the following scores for the qualitative variables used in our model: etiology (HCC = 1, cirrhosis = 2, tumors = 2, other = 4), prior PVT (yes = 2, no = 1), PV obstruction simplified (complete = 2, incomplete = 1), and superior mesenteric vein (complete = 2, incomplete = 1). The score used is the product of the aforementioned factors (*model 1*).

**Figure 7:**
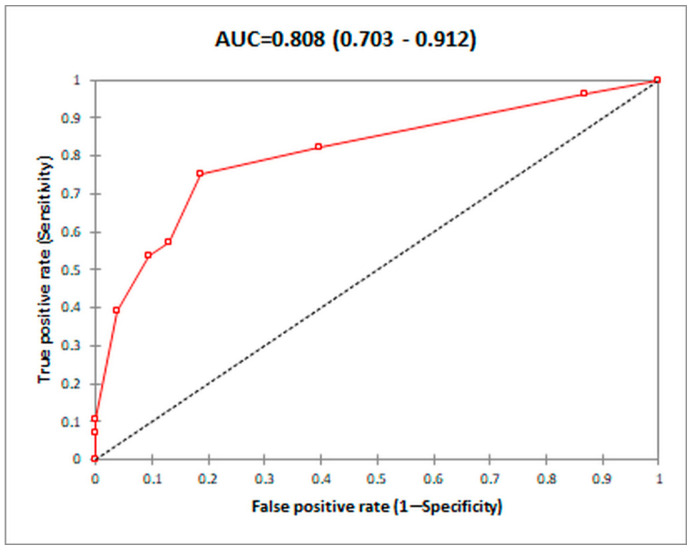
Area under the curve (AUC) operator for the prediction of portal vein thrombosis using model 1.

Calculating the 95% confidence interval for the area under the ROC curve (AUC) for the score involving four variables, we obtained the following results: AUC = 0.808 (CI 95%, 0.703–0.912). Since the confidence interval does not include the neutral value, 0.5, we can conclude that the AUC value differs significantly from 50%, so using the ROC curve we can determine a threshold value to separate the two categories of patients, with or without PC (Figure 7).

Calculating the sensitivities and specificities for various thresholds of the proposed score, we found several values of interest for the detection of PC (Table 6), the best being a threshold value >2, for which the sensitivity (80.77%) is greater than 70%, but the specificity (73.24%) is smaller than 75%, resulting in a Youden index of 0.540.

**Figure 8:**
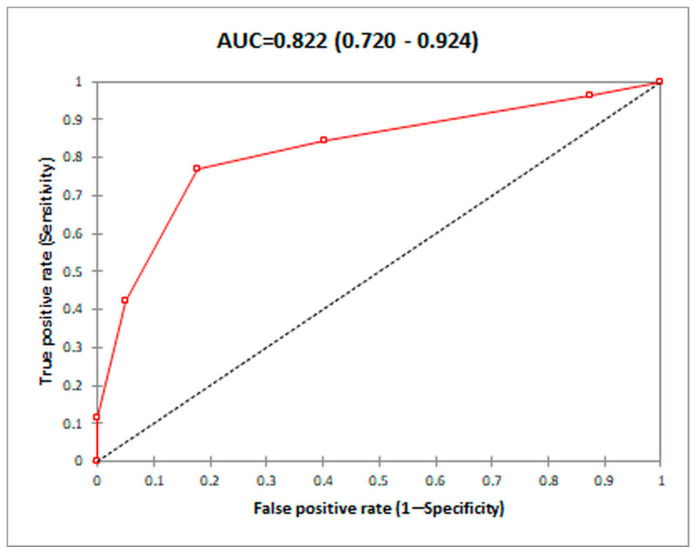
Area under the curve (AUC) operator for the prediction of portal vein thrombosis using model 2.

Because of the suboptimal specificity of the previous score, we tried to improve the specificity by introducing a fifth variable, Prior NSBB use, coded yes = 1, no = 2 (model 2). By calculating the 95% confidence interval for the area under the ROC curve (AUC) for the score involving five variables, we obtained the following results: AUC = 0.822 (CI 95%, 0.720–0.924), slightly better than in model 1 (Figure 8). Since the confidence interval does not include the neutral value, 0.5, we can conclude that the AUC value differs significantly from 50%.

By calculating the sensitivities and specificities for the thresholds of the new score, we found several values of interest for the detection of PC (Table 7), the best being a threshold value >4, for which both the sensitivity (76.92%) and specificity (82.39%) are greater than 75%, resulting in a Youden index of 0.593 (better than in model 1).

## 4. Discussion

In our study, the presence of PC was noted in 14.8% of cases with PVT. The presence of PC was estimated in several studies at between 17–60% [6,25,32,33,34,35,36,37], but a marked heterogeneity of the studies regarding etiology, inclusion, diagnosis criteria, and proportion for complete/incomplete obstruction, exclusion of patients with PC at the moment of the diagnosis, and the rate of SMV thrombosis was noted. Some studies have shown that a significant proportion of patients had already PC from the first diagnosis of PVT; in a study of 131 patients followed up to 6 weeks, 50.4% had PC from the beginning, and in 6.9% of cases PC had appeared during the follow-up [32], while in another study including 62 patients, 26.2% had PC at the moment of the diagnosis of PVT and another 26.1% developed PC during a median follow-up of 7.7 months [37]. In our study, 24 cases of PC were present from the initial diagnosis (12.7% of total PVT), and four cases appeared during a follow-up of 12 months (2.1%). The risk of PC appearance during follow-up seems lower in cases of partial obstruction of the portal vein; in a study, 14% of patients with partial PVT developed complete PVT during 2 years of follow-up, but no PC was seen [33].

The risk of PC was not related in our study to age and gender; however, age was a risk factor in univariate analysis but not in multivariate analysis. Regarding the etiology of PVT, the rate of PC was lower in patients with tumors (6.8%) and cirrhosis (12.7%), and higher in patients with other causes of PVT (thrombophilia, acute and chronic pancreatitis, other inflammatory diseases, myeloproliferative disorders, and idiopathic), at 45.2%. The PC rate was lower in HCC (4.8%) and higher in non-HCC tumors (15.8%), but the difference was not statistically significant. In multivariate analysis, the statistically significant difference was recorded only for other causes of PVT (thrombophilia, inflammation, myeloproliferative disorders, and idiopathic TVP) versus tumors, and cirrhosis-related PVT. The appearance of PC in adults with PVT is related to portal obstruction and the subsequent loss of 2/3 of blood flow. In the acute phase, dilation of the hepatic artery occurs and tries to stabilize the blood flow; after several days, rapid dilation of collateral vessels around the portal vein occurs (“venous rescue”), and in most cases, the development of collateral circulation (including eso-gastric varices), together with partial or total recanalization redesigns the local hemodynamic [2,13]. In this setting, we may expect a lower prevalence of PC in cirrhosis and cirrhosis-associated HCC, as stasis may prevent the development of periportal collaterals [26], but also due to prior alterations of portal circulation, and because of collateral circulation; however, in our study, the difference was not significant statistically. Some studies have also shown that in PVT without liver disease, the prevalence of PC may be higher [26].

Prior PVT was noted in 17.9% of patients with PC and 5% of patients with no PC (OR 5.40, 95% CI 1.51–19.27, *p* = 0.009). Patients with prior PVT have a long evolution of the thrombosis and therefore the probability of PC has increased over time. No prior NSBB use was associated with a higher probability of PC (OR = 2.90, 95%CI (1.21–6.96, *p* = 0.002), although in multivariate analysis, statistical significance was not attained (*p* = 0.087), because of the effect of etiology. We may speculate that the lower probability of PC in patients with prior use of NSBBs may be related to the hemodynamic effects of NSBBs toward portal and systemic circulation, but also the collateral circulation in cirrhosis with already altered local circulation may be involved. In a review article, NSBBs were mentioned as a potential cause for an increased prevalence of PVT in cirrhosis, by decreasing portal blood flow and portal pressure [3]. We noted no relation between PC and prior use of anticoagulant therapy and prior UGIB. No relation between the PC risk and acute onset or the onset with UGIB was noted.

In patients with SMV thrombosis, the presence of PC was correlated with distal SMV extension of the thrombus (OR = 10.33, 95%CI 1.67–64, *p* = 0.012). No relation to SV thrombosis was noted. The presence of PC was related to the degree of PV obstruction, being noted in 24.1% of patients with complete obstruction, 7.7% of patients with partial obstruction, and 5% of patients with one-branch obstruction (OR complete vs. incomplete obstruction, 4.41, 95%CI 1.56 to 12.49, *p* = 0.005). Although the rate of PC was higher than in our study, another study also showed a higher PC rate in patients with complete obstruction (90.9%), versus 28.9% in those with partial obstruction [11]. Superior mesenteric vein thrombosis (SMVT) was present in 21/60 patients and splenic vein thrombosis (SVT) in 3/60 patients; 35% were asymptomatic [11].

Our composite score (model 2), which includes etiology, prior diagnosis of PVT, prior NSBB use, complete versus incomplete PVT, and complete versus proximal SMV thrombosis, has demonstrated good performance for the prediction of PC, with an AUC of 0.822 and an estimated sensitivity of 76.92% and specificity of 82.39% at a cut-off value of 4, being slightly better than model 1 without prior NSBB use (AUC 0.808, sensitivity of 80.77 and specificity of 73.24% at a cutoff of 2). Although several studies have analyzed the risk factors for PC development, there is no study yet which has proposed a composite score for the prediction.

There are some limitations of this study. The retrospective design, together with the lack of compliance for follow-up in approximately 1/3 of cases, did not permit an accurate follow-up to assess the later appearance of the cavernoma. As in other studies, the group of patients with PC was not very large (28 cases); most cases (85.7%) of cavernoma appeared from the moment of the diagnosis, and in 21 cases, there was no proper CT scan or MRI characterization of the SMV invasion.

## 5. Conclusions

In our study, the presence of portal cavernoma was noted in 14.8% of PVT cases, lower than in other studies. The rate of PC was higher in patients with complete versus incomplete portal vein obstruction (24.1 versus 7.7%), in patients with PVT related to inflammation, thrombophilia, and unknown etiology versus those related to tumors non-HCC, HCC, and cirrhosis (45.2 versus 15.8 versus 12.7 versus 4.8%), and in patients with prior PVT (17.9 versus 5% for those with no previous PVT); in patients with prior NSBB use the prevalence was 9.2% and in those without prior NSBB use was 22.5%. In patients with SMV thrombosis, the presence of a distal extension of the thrombus was associated with a greater probability of PC. Our composite score, including etiology, prior diagnosis of PVT, prior NSBB use, complete versus incomplete PVT, and complete versus proximal SMV thrombosis, has demonstrated good performance for the prediction of PC, with an AUC of 0.822 (model 2) and an estimated sensitivity of 76.92% and specificity of 82.39% at a cut-off value of 4.

## Figures and Tables

**Table 2:** PVT patient’s characteristics.

Age (Mean ± Std Dev)	63.2 ± 11.3 Years
<<40/40–59/60–79/>80 (%)	4.2/33.3/58.2/4.2
M/F (%M)	136/53 (72.0%)
Etiology (%)	
Tumors	54.5
HCC	44.4
Gastric	3.2
Pancreatic	3.2
Colorectal	1.1
Other tumors	2.6
Cirrhosis	29.1
Local inflammation	5.8
Chronic pancreatitis	3.7
Myeloproliferative neoplasms	1.1
Sleeve gastrectomy	0.5
Unknown	4.3
Cavernoma (%)	28 (14.8)
Cirrhosis etiology (%)	
Alcohol	67.7%
Hepatitis B virus	11.8%
Hepatitis C virus	15.5%
Biliary	1.8%
No data	11.5%
Prior PVT/PC, prior treatment (%)	
PVT	13 (6.9)
PC	1 (0.5)
UGIB	8 (4.2)
NSBBs	109 (57.7)
Anticoagulants	20 (10.6)
Onset (%)	
Acute pain	36 (19.0)
UGIB	33 (17.4)
Anticoagulant therapy introduced (%)	54 (28.6)
Imaging procedures performed/visible thrombus (%)	
Ultrasound	88.9/83.3
CT scan	75.1/86.6
MRI	18.5/91.4
CEUS	25.9/100
Thrombus extension and aspect	
Portal (complete/incomplete/one branch)	87/78/20/3
SMV (proximal/complete/no/unevaluated)	34/8/126/21
SV (proximal/complete/no/unevaluated)	52/7/109/21
Endoscopy	108
No E varices (%)	21 (19.4)
Small E varices (%)	44 (40.7)
Large E varices (%)	43 (39.8)
G varices (%)	3 (2.8)

Abbreviations: E = esophageal, G = gastric, PVT = portal vein thrombosis, PC = portal cavernoma, NSBBs = non-selective beta-blockers, UGIB = upper gastrointestinal bleeding, CT = computed tomography, MRI = magnetic resonance imaging, CEUS = contrast-enhanced ultrasound, SMV = superior mesenteric vein, SV = splenic vein.

**Table 3:** Characteristics of PC patients and those without PC.

	PC No = 28	No PC No = 161	*p*-Value
Age (mean ± std dev)	57.4 ± 16.5	64.2 ± 9.9	*0.003*
M/F	18/10	118/43	0.330
Etiology (%)			
Tumors excluding HCC	3 (10.7)	16 (9.9)	*<0.001*
HCC	4 (14.3)	80 (49.7)	
Cirrhosis	7 (25)	48 (29.8)	
Other	14 (50)	17 (10.6)	
Prior PVT/PC, prior treatment (%)			
Prior PVT	5 (17.9)	8 (5)	*0.020*
Prior UGIB	2 (7.1)	6 (3.7)	0.526
Prior NSBBs	10 (35.7)	99 (61.5)	*0.013*
Prior anticoagulant therapy	4 (14.3)	16 (9.9)	0.493
Onset (%)			
Acute pain	4 (14.3)	32 (19.9)	0.489
UGIB	5 (17.9)	28 (17.4)	0.952
Thrombus extension and aspect			
Portal (complete/incomplete/ one branch)	21/6/1	66/72/19	*<0.001*
SMV (proximal/distal/no/unevaluated)	3/4/19/2	31/4/107/19	*0.015*
SV (proximal/distal/no/unevaluated)	5/1/20/2	47/6/89/19	0.357
Endoscopy (%)			
No varices	5 (27.8)	16 (17.8)	0.411
Small varices	5 (27.8)	39 (43.3)	
Large varices	8 (44.4)	35 (38.9)	

Abbreviations: Std = standard deviation, PVT = portal vein thrombosis, PC = portal cavernoma, NSBBs = non-selective beta-blockers, UGIB = upper gastrointestinal bleeding, SMV = superior mesenteric vein, SV = splenic vein. Italicized fonts represent statistically significant *p*-values.

**Table 4:** Risk factors for portal cavernoma in univariate and multivariate analysis.

	Crude OR (95% CI)	*p*-Value	Adjusted OR (95% CI)	*p*-Value
Age	0.96 (0.92–0.99)	*0.015*	0.98 (0.94–1.03)	0.437
Etiology				
Cirrhosis vs. HCC	2.50 (0.67–9.37)	0.174	2.09 (0.47–9.39)	0.336
Tumors (no HCC) vs. HCC	3.75 (0.75–18.63)	0.106	1.48 (0.23–9.67)	0.683
Others vs. HCC	14.22 (4.09–49.40)	*<0.001*	7.01 (1.61–30.53)	*0.010*
Prior PVT	5.40 (1.51–19.27)	*0.009*	6.48 (1.14–36.89)	*0.035*
No prior NSBBs	2.90 (1.21–6.96)	*0.017*	3.00 (0.85–10.58)	0.087
Portal vein obstruction				
Complete vs. incomplete	4.41 (1.56–12.49)	*0.005*	4.66 (1.37–15.84)	*0.014*
Single branch vs. incomplete	0.72 (0.08–6.58)	0.773	1.09 (0.10–12.00)	0.941
SMV obstruction				
No obstruction vs. proximal	1.83 (0.51–6.61)	0.353	2.72 (0.63–11.79)	0.182
Complete vs. proximal	10.33 (1.67–64.00)	*0.012*	6.40 (0.63–64.72)	0.116

Abbreviations: OR = Odds Ratio, HCC = hepatocellular carcinoma, PVT = portal vein thrombosis, NSBBs = non-selective beta-blockers, SMV = superior mesenteric vein. Italicized fonts represent statistically significant *p*-values.

**Table 5:** Risk factors for portal cavernoma in multivariate analysis.

	Adjusted OR (95% CI)	*p*-Value
Etiology		
Cirrhosis vs. HCC	2.12 (0.50–9.01)	0.307
Tumors (excluding HCC) vs. HCC	3.16 (0.59–17.00)	0.180
Others vs. HCC	13.18 (3.48–49.89)	*0.000*
Prior PVT	5.10 (0.90–28.96)	0.066
Portal vein obstruction		
Complete vs. incomplete	3.99 (1.25–12.74)	*0.020*
Single branch vs. incomplete	0.82 (0.08–8.56)	0.871
Superior mesenteric vein obstruction		
No obstruction vs. proximal	2.52 (0.60–10.59)	0.207
Complete vs. proximal	5.54 (0.62–49.08)	0.124

Abbreviations: OR = Odds Ratio, HCC = hepatocellular carcinoma, PVT = portal vein thrombosis. Italicized fonts represent statistically significant *p*-values.

**Table 6:** Values of interest for sensitivity and specificity for different score thresholds (model 1).

Threshold	Sensitivity	Specificity	Sensitivity + Specificity	Accuracy
1	92.31%	23.94%	116.25%	34.52%
2	80.77%	73.24%	154.01%	74.40%
4	50.00%	92.25%	142.25%	85.71%
8	19.23%	100.00%	119.23%	87.50%
16	3.85%	100.00%	103.85%	85.12%
32	0.00%	100.00%	100.00%	84.52%

**Table 7:** Values of interest for sensitivity and specificity for different score thresholds (model 2).

Threshold	Sensitivity	Specificity	Sensitivity + Specificity	Accuracy
1	96.15%	12.68%	108.83%	25.60%
2	84.62%	59.86%	144.47%	63.69%
4	76.92%	82.39%	159.32%	81.55%
8	42.31%	95.07%	137.38%	86.90%
16	11.54%	100.00%	111.54%	86.31%
32	0.00%	100.00%	100.00%	84.52%

## Data Availability

The data used to support the findings of this study are available from the corresponding author upon request.
